# Adjuvant 5FU plus levamisole in colonic or rectal cancer: improved survival in stage II and III

**DOI:** 10.1054/bjoc.2001.2117

**Published:** 2001-11

**Authors:** B G Taal, H Van Tinteren, F A N Zoetmulder, on behalf of the NACCP group

**Affiliations:** Departments of Gastroenterology, Medical Oncology, Statistics and Surgery, Netherlands Cancer Institute/Antoni van Leeuwenhoek Hospital, Amsterdam

**Keywords:** colonic cancer, rectal cancer, adjuvant therapy

## Abstract

Based on the first favourable results of adjuvant therapy of 5FU plus levamisole in Dukes C colonic cancer in 1990, we conducted a prospective trial. 1029 patients were randomised to receive one year 5FU plus levamisole or no further treatment following curative surgery for stage II or III colon (*n* = 730) or rectal cancer (*n* = 299). 45% were in stage II and 55% in stage III. With a median follow-up of 4 years and 9 months a significant reduction in odds of death (25%, SD 9%, *P* = 0.007) was observed for those with adjuvant treatment (65% at 5 year) compared to the observation group (55%). Improved relative survival was present in stage III (56% vs 44%), and in stage II patients (78% vs 70%). In rectal cancer a non-significant difference in disease-free or overall survival was observed. Distant metastases developed in 76%, while local recurrence alone occurred in 14%. An early start of adjuvant treatment (< 4 weeks) did not affect results. Compliance to 5FU plus levamisole was 69%. Severe toxicity did not occur. In conclusion, one year 5FU plus levamisole was of benefit in stage II and III colonic cancer; in rectal cancer a significant positive effect could not be demonstrated. © 2001 Cancer Research Campaign  http://www.bjcancer.com


*British Journal of Cancer* (2001) **85**(10), 1437–1443
© 2001 Cancer Research Campaign
doi: 10.1054/ bjoc.2001.2117, available online at http://www.idealibrary.com on IDEAL

http://www.bjcancer.com

### Adjuvant 5FU plus levamisole in colonic or rectal cancer: improved survival in stage II and III

**BG Taal[1,2], H Van Tinteren[3] and FAN Zoetmulder [4] on behalf of the NACCP group***

Departments of [1]Gastroenterology, [2]Medical Oncology, [3]Statistics and [4]Surgery, Netherlands Cancer Institute/Antoni van Leeuwenhoek Hospital, Amsterdam

**Summary** Based on the first favourable results of adjuvant therapy of 5FU plus levamisole in Dukes C colonic cancer in 1990, we conducted a prospective trial. 1029 patients were randomised to receive one year 5FU plus levamisole or no further treatment following curative surgery for stage II or III colon ($n$ = 730) or rectal cancer ($n$ = 299). 45% were in stage II and 55% in stage III. With a median follow-up of 4 years and 9 months a significant reduction in odds of death (25%, SD 9%, $P$ = 0.007) was observed for those with adjuvant treatment (65% at 5 year) compared to the observation group (55%). Improved relative survival was present in stage III (56% vs 44%), and in stage II patients (78% vs 70%). In rectal cancer a non-significant difference in disease-free or overall survival was observed. Distant metastases developed in 76%, while local recurrence alone occurred in 14%. An early start of adjuvant treatment (< 4 weeks) did not affect results. Compliance to 5FU plus levamisole was 69%. Severe toxicity did not occur. In conclusion, one year 5FU plus levamisole was of benefit in stage II and III colonic cancer; in rectal cancer a significant positive effect could not be demonstrated. © 2001 Cancer Research Campaign http://www.bjcancer.com

**Keywords**: colonic cancer; rectal cancer; adjuvant therapy

Until 1990 a broad consensus existed both in the surgical and medical communities in the Netherlands that there was no role for adjuvant chemotherapy in the standard treatment of colon or rectal cancer. Although 5FU-based adjuvant therapy had been shown to produce a small survival benefit in a meta-analysis in 1988, this benefit was found to be too small to warrant routine use of this toxic therapy (Buyse et al, 1988). In 1990, Moertel et al published the 3-year results of the Intergroup Study, showing for the first time a clinically important survival benefit of 1 year adjuvant therapy with 5FU plus levamisole. This trial confirmed the promising results of the prior NCTG trial (Laurie et al, 1989). The American Consensus meeting adopted shortly afterwards 5FU plus levamisole as standard therapy in stage III colon cancer (NIH, 1990). In the Netherlands, however, many clinicians remained in doubt for a number of reasons. Levamisole had previously been found not effective in colorectal cancer, neither in advanced, nor in the adjuvant situation. It was difficult to understand why 5FU, which has such a limited effect in advanced disease, would on itself have such an enormous impact on survival in the adjuvant setting. The survival benefit in stage III but equivocal in stage II, seemed to contradict the general assumption that adjuvant chemotherapy is the more effective as the microscopic tumour residue is smaller.

Consequently, it was felt that more evidence was needed to evaluate the effect of 5FU plus levamisole. Assuming that micrometastases would have similar sensitivity to systemic treatment whether originating from colon or rectal cancer, both tumour sites were included in the present trial. Randomisation was, however, stratified for colon and rectum to guarantee balance for treatment comparisons. For this study the Netherlands Adjuvant Colorectal Cancer Project was established, a cooperative group including 52 hospitals in the Netherlands, treating approximately 60% of colorectal cancer cases in the country.

In this paper we present results of an analysis at a median follow-up of 4 years and 9 months.

### MATERIALS AND METHODS

### Patient selection

Patients potentially curatively resected for stage II (Dukes B) or stage III (Dukes C) adenocarcinoma of the colon or rectum and a WHO performance 0–2 were eligible. Perforation, concomitant chronic inflammatory bowel disease or familial polyposis coli, age over 75 years, or previous malignancies were exclusion criteria. In addition, bone marrow function, renal clearance and liver tests had to be normal. Informed consent was obtained either orally or written according to the regulations of each participating hospital. Also a video tape with patient information presented by FAN Zoetmulder was available.

*Received 17 April 2001*
*Revised 26 July 2001*
*Accepted 30 July 2001*

*Correspondence to:* BG Taal

*****NACCP working party:
C. J. H. v.d. Velde MD. Ph.D, Department of Surgery, Leiden University Medical Center; J. Jeekel MD, PhD, Department of Surgery, University Hospital Rotterdam, Dijkzigt Hospital; T. Kok MD, PhD, Department of Medical Oncology, University Hospital Rotterdam, Dijkzigt Hospital; C.W. Taat MD, Ph.D., Department of Surgery, Academic Medical Center, Amsterdam; C.H.N. Veenhof MD, Ph.D, Department of Medical Oncology, Academic Medical Center, Amsterdam; S. Meyer MD, PhD, Department of Surgery, Free University, Amsterdam; Th. Wiggers, MD, PhD, Department of Surgery, Daniel den Hoed Cancer Center, Rotterdam; J.A. Roukema MD, PhD, Department of Surgery, Elisabeth Hospital, Tilburg; M.Eliel MD, Comprehensive Cancer Center Mid and North Netherland, Utrecht; P.W. de Graaf, MD, PhD, Department of Surgery, Reinier de Graff Hospital, Delft; E.J.Th. Rutgers MD, PhD, Department of Surgery, Netherlands Cancer Institute/Antoni van Leeuwenhoekhuis, Amsterdam; O. Dalesio Sc, Department of Biometrics, Netherlands Cancer Institute/Antoni van Leeuwenhoek Hospital, Amsterdam.

### Trial design

This study is a randomised phase III trial comparing surgery alone with surgery plus adjuvant treatment of one year 5-FU plus levamisole (the positive arm in the Moertel study). Patients from 52 hospitals all over the Netherlands were randomised between the end of 1990 and February 1996. After resection patients were randomly assigned by telephone in the Netherlands Cancer Institute Trial Office, only after checking the eligibility criteria. Patients were stratified according to site (colon or rectum), stage (II or III), additional adjuvant radiotherapy (yes or no) and for participating institution.

Treatment had to start within 8 weeks after resection. 5-FU was administered as a push injection (450 mg $m^{-2}$) daily for 5 consecutive days. From day 28 onwards 5-FU was given once weekly in the same dose and continued for 48 weeks. Guidelines for postoperative adjuvant irradiation (rectal cancer T3 and/or N1) varied slightly among centres. Since the randomisation procedure was per centre this was not a contraindication for participation. In such a case the 5-day loading dose of 5FU was administered prior to the start of radiotherapy (50 Gy in 5 weeks) in order to avoid excessive toxicity; the further weekly doses from week 4 onwards were given partially concomitant with the radiotherapy. Concurrent to the 5-FU loading dose, levamisole was started orally in 3 daily doses of 50 mg during 3 days and repeated every 2 weeks for 52 weeks.

Toxicity was graded according to the WHO criteria. In case of grade 3 or 4 mucositis, diarrhoea or bone marrow depression the 5-FU dose was reduced by 20% after complete recovery. In case of grade 3 or 4 neurotoxicity the levamisole dose was reduced by 50%.

Patients in both groups were seen at least twice a year during the first 2 years after surgery and at least once a year thereafter for a minimum of 5 years. These evaluations consisted of medical history and physical examination. In addition, endoscopy of the remaining colon, ultrasound of the liver and a chest X-ray every year was suggested.

The protocol was approved by the Dutch Clinical Research Foundation (CKVO) and could make use of the system of Comprehensive Cancer Centres in the Netherlands, which provides specialist support and data management to all hospitals treating cancer patients. Local Medical Ethical Committees approval of all participating hospitals was required before entering patients.

### Statistical analysis

Time to overall mortality, calculated from randomisation, was the main endpoint of the study. Time to recurrence or metastatic disease was also measured. To be able to detect a reduction in mortality from an estimated 50% to an overall 5 year mortality of 42% with a power of 90% (2-tailed test, $P < 0.05$) at least 2000 patients were needed.

Blinded interim analyses were performed after 1 and 2 years median follow-up and discussed within an independent monitoring committee.

The trial was closed after 5 years, when 1029 patients were enrolled. It was decided to stop the trial in February 1996 when more evidence from other trials became available in favour of adjuvant treatment, and the ultimate goal to include 2000 patients was not expected to be reached within a couple of years. Median actuarial follow-up was 4 years and 9 months. Recurrence-free and overall survival curves were constructed using the Kaplan–Meier technique and compared by the log-rank test. Heterogeneity of the treatment effect between different levels of potentially prognostic factors (test for interaction) was tested by the proportional hazard analysis. Data were analysed according to the allocated treatment only (intention-to-treat). Compliance was calculated from start of treatment until stop of treatment. For this particular analysis patients that recurred ($n = 46$) or died ($n = 4$) within the year of adjuvant treatment, were censored at time of event.

### Analysis within specific subgroups

Because the targeted sample size was not reached, subgroup analysis should be interpreted with caution. Recurrence-free and overall survival of 5FU plus levamisole versus control were compared within the subgroups of stage II and III disease, colon and rectal cancer, gender, age and performance status. In rectal cancer the treatment comparison was made additionally for whether or not the patients had been treated with postoperative irradiation. For each subgroup the ratio of the failure rates of 5FU plus levamisole versus control is plotted as a black square (as applied in Figures 4 and 5). Its size is proportional to the amount of information concerned, so that subgroups with more patients and events are represented by larger squares. The vertical dotted line corresponds to ratios of 1.0 that appear when results of treatment and control are similar. Ranges smaller than 1.0 (to the left of the vertical line) indicate a beneficial effect of 5FU plus levamisole, while ranges larger than 1.0 correspond to an adverse effect of adjuvant treatment. A confidence interval (99% for each subgroup; 95% for 5FU plus levamisole versus control overall) is depicted as a horizontal line through each square. If the horizontal line crosses the vertical line, the confidence interval contains the ratio of 1.0 and the comparison of treatments in a particular subgroup is not significant at 1%. This level of significance was chosen to take into account the increased probability of error due to multiple testing. To statistically test whether the effect of treatment was different between levels of subgroups, a test for interaction was performed.

### RESULTS

A total of 1029 patients entered the study (Figure 1): 515 were randomly assigned to no further treatment and 514 to the adjuvant therapy of 5-FU plus levamisole. Among the 514 patients assigned to adjuvant treatment 49 patients (9.5%) did not start treatment: 35 patients refused treatment after randomisation, 3 patients (6%) had postoperative complications, 4 patients (8%) had residual disease and 7 patients (14%) had various other reasons. Of the patients that did not start 36 had colon and 13 rectal cancer. Not starting was irrespective of the stage of disease; however, complications as reason for not starting 5FU plus levamisole were only seen in stage III disease. On review, 13 patients did not meet the eligibility criteria of the protocol: 6 patients were over the age of 75 years, 2 patients had prior cancer, 4 patients appeared to harbour concurrent liver metastases, and in one patients no malignancy after the final histological report of the resection material.

Median time from surgery to randomisation was 5 weeks. In 79 patients the interval was more than 6 weeks (median 8 weeks). The clinical characteristics (Table 1) were well balanced between the 2 study arms. A majority (58%) of the patients was aged 60 years or older, and the performance status was usually good. A minority

 © 2001 Cancer Research Campaign

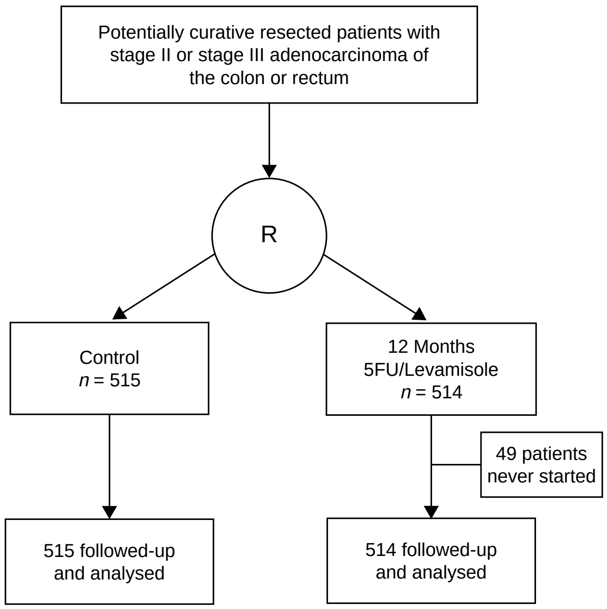

**Figure 1** Trial profile

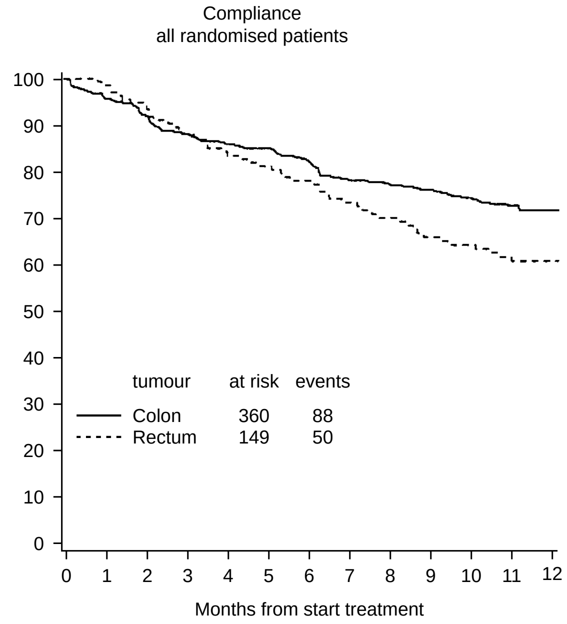

Numbers at risk

| | | | | | | | | | | | | | |
|---|---|---|---|---|---|---|---|---|---|---|---|---|---|
| Colon | 360 | 308 | 293 | 277 | 268 | 263 | 251 | 237 | 232 | 227 | 216 | 210 | 206 |
| Rectum | 149 | 134 | 125 | 116 | 109 | 106 | 101 | 93 | 85 | 79 | 76 | 70 | 68 |

**Figure 2** Compliance for all randomised patients

**Table 1** Initial clinical characteristics of all patients

| Characteristic | Randomisation arm: Surgery alone (*n* = 515) | | Randomisation arm: 5FU plus Levamisole (*n* = 514) | | Total (*n* = 1029) | |
|---|---|---|---|---|---|---|
| | *n* | % | *n* | % | *n* | % |
| Gender | | | | | | |
| male | 298 | 56.1 | 301 | 58.6 | 590 | 57.3 |
| female | 226 | 43.9 | 213 | 41.4 | 439 | 42.7 |
| Localisation tumour | | | | | | |
| colon | 365 | 70.9 | 365 | 71.0 | 730 | 70.9 |
| rectum | 150 | 29.1 | 149 | 29.0 | 299 | 29.1 |
| Stage classification | | | | | | |
| stage II | 235 | 45.6 | 233 | 45.3 | 468 | 45.5 |
| stage III | 280 | 54.4 | 281 | 54.7 | 561 | 54.4 |
| Age | | | | | | |
| < 60 y | 211 | 41.0 | 215 | 41.8 | 426 | 41.4 |
| 60–65 y | 136 | 26.4 | 143 | 27.8 | 279 | 27.1 |
| > 65 y | 168 | 32.6 | 156 | 30.4 | 324 | 31.5 |
| Age | | | | | | |
| mean age (SD) | 59.6 (9.8) | | 59.9 (9.4) | | 59.8 (9.6) | |
| median age (range) | 61.0 (27–76) | | 61.5 (27–80) | | 61.0 (27–80) | |
| Performance status | | | | | | |
| WHO 0 | 445 | 86.4 | 430 | 83.7 | 875 | 85.0 |
| WHO 1 | 70 | 13.6 | 84 | 16.3 | 154 | 15.0 |
| Radiotherapy | | | | | | |
| colon (distal sigmoid) | 13 | 3.6 | 7 | 1.9 | 20 | 2.7 |
| rectum | 88 | 58.7 | 79 | 53.0 | 167 | 55.9 |

was in stage II (45%) compared to 55% stage III. Radiotherapy was applied mainly in rectal cancer (55 in stage II and 112 in stage III) and only 20 patients (7 in stage II and 13 in stage III) of the colonic cancer group (sigmoid tumours). In all cases it was balanced over both the trial arms.

As illustrated in Figure 2 compliance was 80% at 6 months and 69% received treatment according to protocol for one year. Discontinuation of adjuvant treatment (both 5FU and levamisole) occurred gradually over time. Stage III patients were significantly more compliant to treatment (absolute difference at 1 year 12% Standard Error 3; logrank $P = 0.01$). Colon cancer patients were more compliant than rectal cancer patients (absolute difference at 1 year 11% Standard Error 2.6; logrank $P = 0.05$). Compliance was also inversely related to age ($P = 0.003$). Reasons for stopping both 5FU and levamisole other than recurrence of disease were most often flu-like symptoms and psychological reasons (55%): this percentage was similar within the colonic and rectal carcinoma subgroups and not related to stage. The overall survival (Figure 3) showed a significant difference in favour of the adjuvant treatment with an estimated 5 year survival of 68% compared to 58% in the control arm and 25% (SD 9%) reduction in odds of death (logrank $P = 0.007$). A total of 365 patients died: 203 in the control group and 162 in the 5FU plus levamisole arm (Table 2). 319 patients died with cancer: 178 in the control group and 141 in the adjuvant treatment arm. Cardiovascular disease was most often reported as the alternative cause of death. Toxic deaths were not reported. Subgroup analysis showed relative survival benefit following adjuvant treatment not only in stage III (27%, SD 11), but also in stage II tumours (19%, SD 15) (Figure 4). Although the prognosis is significantly different for each stage, the size of reduction in odds of death by treatment was similar (Figure 5).

Despite the inclusion in the trial within 8 weeks the actual start of treatment was sometimes delayed ($n = 34$ in colonic cancer and $n = 7$ in rectal cancer) by the extensive discussions with the patient and the family. Within the treatment arm an early start of 5FU plus levamisole (within 4 weeks) did not affect the results compared to the patients that started after 4 weeks with the adjuvant treatment.

In rectal cancer, although the effect was in the same direction, the difference (Figure 6) was only small with a wide confidence interval. Test for interaction, however, did not show significant heterogeneity ($P = 0.133$). Other factors such as gender, age or

© 2001 Cancer Research Campaign performance status did not affect the outcome (Figure 5). Radiation therapy, applied in rectal or distal sigmoid cancer, appeared not to be related to the effect of systemic adjuvant treatment. 68 patients (46%) completed the one year therapy course, recurrent disease appeared in 10% and the remaining 44% stopped treatment due to toxicity. In colonic cancer 58% completed the full course, 9% stopped due to recurrence and 33% for toxicity.

Recurrence-free survival was also significantly better in the 5FU plus levamisole arm with a 21% (SD 8%) relative reduction in the risk of failing. In this regard the impact of the aforementioned factors were similar (Figure 7). Distant metastases were the main cause of treatment failure (76%), while local recurrence alone occurred in 14%; 10% of patients died of non-malignant causes.

**Table 2** The event status of all patients

| | **Randomisation arm** | | | | | |
|---|---|---|---|---|---|---|
| **Outcome** | **Surgery alone (*n* = 515)** | | **5FU plus Levamisole (*n* = 514)** | | **Total (*n* = 1029)** | |
| | ***n*** | **%** | ***n*** | **%** | ***n*** | **%** |
| Alive without recurrence | 260 | 50.5 | 298 | 58.0 | 558 | 54.2 |
| Alive with recurrence | 52 | 10.1 | 54 | 10.5 | 106 | 10.3 |
| Dead without recurrence | 25 | 4.8 | 21 | 4.1 | 46 | 4.5 |
| Dead with recurrence | 178 | 34.6 | 141 | 27.4 | 319 | 31.0 |

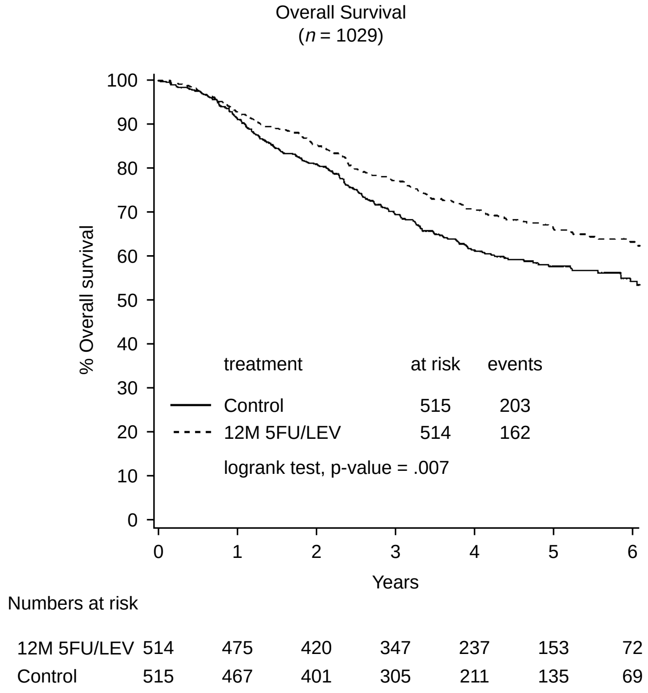

**Figure 3** Overall survival in all patients with colorectal cancer (*n* = 1029) according to treatment

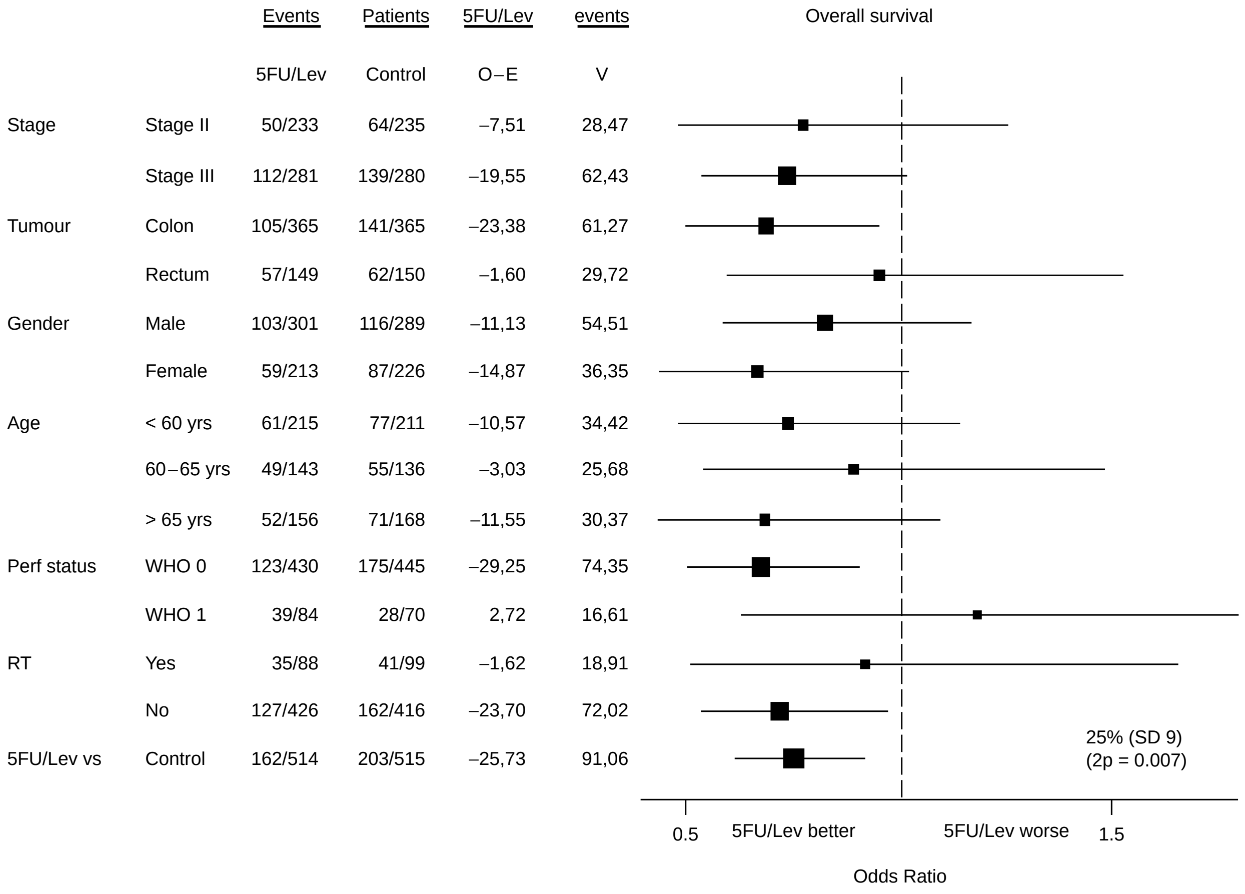

**Figure 5** Overall survival in subgroup analysis according to treatment with observed minus expected ratio (O–E), its variance (V) and the number of events

 *© 2001 Cancer Research Campaign*

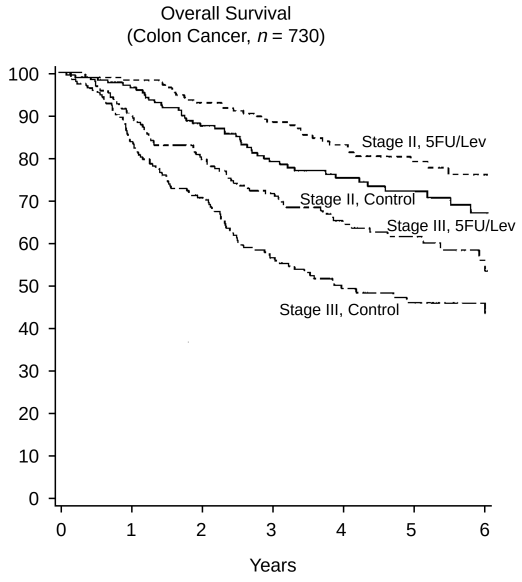

**Figure 4** Overall survival in the patients with colonic cancer (*n* = 730) according to stage

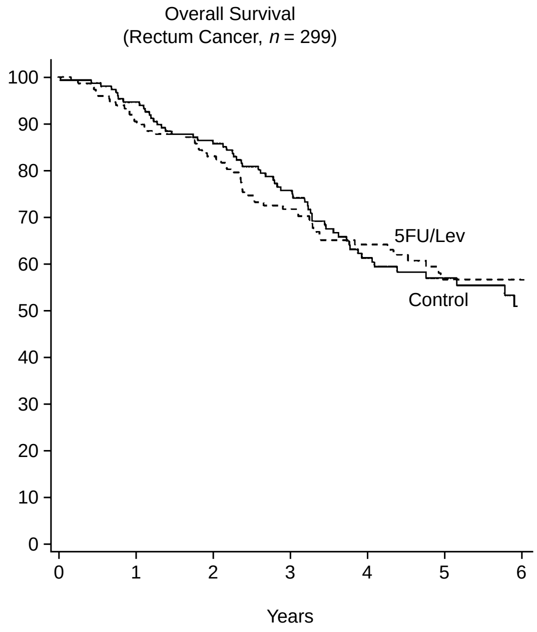

**Figure 6** Overall survival in patients with rectal cancer (*n* = 229) according to treatment

| | | Events 5FU/Lev | Patients Control | 5FU/Lev O-E | events V |
|---|---|---|---|---|---|
| Stage | Stage II | 68/233 | 82/235 | –7,82 | 37,46 |
| | Stage III | 148/281 | 173/280 | –21,50 | 79,79 |
| Tumour | Colon | 145/365 | 175/365 | –22,61 | 79,69 |
| | Rectum | 71/149 | 80/150 | –3,98 | 37,73 |
| Gender | Male | 138/301 | 144/289 | –10,26 | 70,15 |
| | Female | 78/213 | 111/226 | –17,11 | 47,11 |
| Age | < 60 yrs | 84/215 | 103/211 | –14,93 | 46,55 |
| | 60–65 yrs | 60/143 | 65/136 | –4,05 | 30,94 |
| | > 65 yrs | 72/156 | 87/168 | –7,91 | 39,53 |
| Perf status | WHO 0 | 174/430 | 221/445 | –28,45 | 98,55 |
| | WHO 1 | 42/84 | 34/70 | 0,61 | 18,82 |
| RT | Yes | 42/88 | 57/99 | –4,43 | 24,60 |
| | No | 174/426 | 198/416 | –21,83 | 92,63 |
| 5FU/Lev vs | Control | 216/514 | 255/515 | –27,30 | 117,49 |

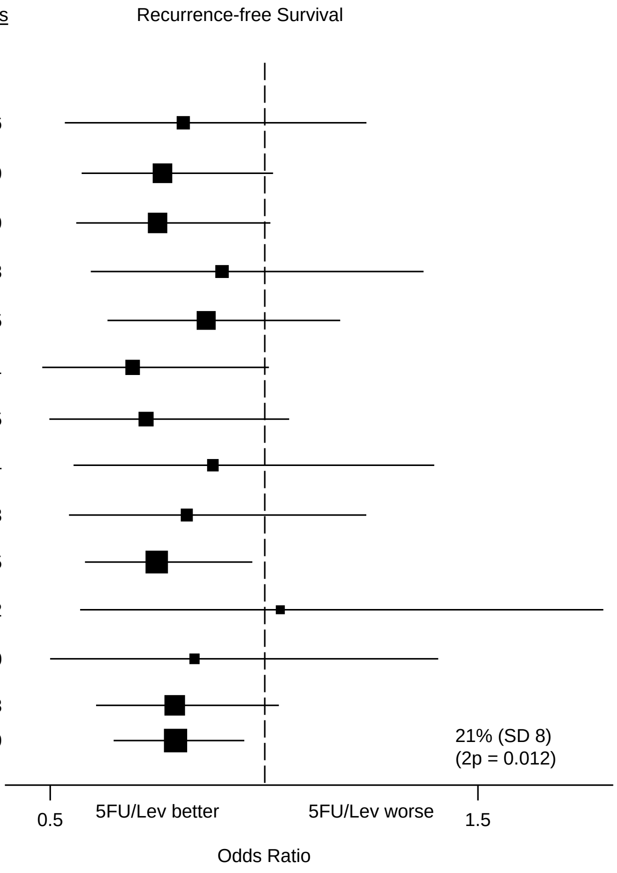

**Figure 7** Recurrence-free survival in subgroup analysis according to treatment with observed minus expected ratio (O–E), its variance (V) and the number of events

### DISCUSSION

The present trial shows a significant reduction in odds of death of 27% (SD 11) in stage III by one year 5FU plus levamisole treatment in agreement with the Intergroup study revealing (Moertel et al, 1990, 1995) a 33% reduction in death rate, compared to surgery alone. In general, adjuvant treatment is recommended to be given as soon as possible after surgery. In normal clinical practice this is often not the case. In our study, however, an early start (within 4 weeks following resection) did not affect survival compared to patients who started after 4 weeks. In later studies 5FU combined with leucovorin has been preferred based on its biochemical interaction (IMPACT, 1995). The pooled analysis of such studies in the IMPACT trial group (including 3 separate trials with a similar design from Canada, Italy and France) revealed also a significant reduction of odds of death by 22% and recurrence by 35% in stage III using 6 months adjuvant 5FU with a high-dose leucovorin (IMPACT, 1995). In addition, similar results were reported by O'Connell et al (1997) applying 6 months 5FU plus low-dose leucovorin. In a randomised study of 1081 patients with colon cancer 5FU plus leucovorin was superior to the MOF scheme (methyl-CCNU, vincristin and 5FU) (Wolmark et al, 1993). Data suggest that 6 months appear to be enough for 5FU plus leucovorin, while 12 months of treatment is needed when 5FU plus levamisole is given (O'Connell, 1998). Therefore, the 6 months scheme of 5FU plus leucovorin is currently recommended as the standard schedule of adjuvant therapy in stage III colonic cancer (O'Connell et al, 1997, O'Connell 1998, Wolmark et al, 1999).

In our study, also in stage II patients ($n$ = 468), a significant benefit was seen in both recurrence-free survival and overall survival leading to an increase from 70 to 78% 5 year survival. The reduction in death rate was 19% (SD 15). Data from the literature in stage II are conflicting in this respect. In the Moertel study (1995) the reduction was 31% in recurrence rate for stage II patients ($n$ = 318) following one year 5FU plus levamisole, but confidence intervals included non-significance. In the aforementioned IMPACT study (1995, 1999) with a large number of stage II patients ($n$ = 1016) adjuvant treatment (6 months 5FU plus leucovorin) did not show a significant effect on 5 year event-free (72% versus 76%) and overall survival (80 versus 82%). The third report dealing with the effect of adjuvant chemotherapy in stage II patients is described by Mamounas et al (1999). They combined and regrouped the data of 1565 patients enrolled in one of 4 NSABP trials and claimed a mortality reduction of 30% in the 5FU plus levamisole and 5FU plus leucovorin group. However, the design of this report was remarkable in its heterogeneity of trials included in this evaluation: 2 trials compared surgery alone with surgery plus adjuvant portal infusion with 5FU or systemic chemotherapy (MOF), while the other trials compared 2 different schemes of adjuvant therapy without a 'surgery alone' arm.

In the present trial rectal cancer was also included. Postoperative radiotherapy was applied in the majority of these patients, and equally divided over both study arms. The beneficial effect of irradiation to prevent local recurrence has been reported by Fisher (NSABP trial R-01) (Fisher et al, 1988), but also negative results have appeared (Treurniet-Donker et al, 1991). In Swedish trials radiotherapy given prior to surgery in a hypofractionated scheme ($5 \times 5$ Gy), resulted in better local control compared to postoperative radiotherapy, or surgery alone (Frycholm et al, 1993; Swedish Rectal Cancer Trial, 1997). A recent Dutch trial of this short-term preoperative scheme induced a decrease in tumour size and number of lymph nodes, but did not lead to down-staging (Marijnen et al, 2001). Data on survival are awaited to emerge. To improve survival in rectal cancer, chemotherapy has been applied, usually a combination of 5FU plus methyl-CCNU, which resulted in a reduction of 29% in death rate and an increase in 5-year survival from 46 to 58% (Krook et al, 1991). However, numbers were small ($n$ = 204), the effect occurring late (after 5 years) and severe delayed toxicity (small bowel obstruction due to radiation fibrosis requiring surgery) occurred in 7%.

In the present study the benefit of adjuvant treatment in rectal cancer was very small and not significant. However, the number of patients ($n$ = 229) was relatively limited and not enough to exclude some activity of adjuvant therapy. Consequently, the result in this group of patients remained unclear. The lack of clear benefit might be explained by different biological tumour behaviour or related to the treatment scheduling. The start of adjuvant treatment was delayed (8–10 weeks from surgery) in 7 patients (4.7%), which compared favourable with 9.3% in colonic cancer. To avoid severe toxicity, the 5 day loading dose of 5FU was given before the start of irradiation, while the subsequent weekly dosages were applied from the end of the radiation scheme (weeks 4–5). Another reason might be the compliance, which was 11% less in rectal carcinoma compared to that in colon cancer. Whether drug dosing was suboptimal, is unknown. Data on dose scheduling are not available from this trial as it was designed to be a large simple trial.

69% of all patients assigned to adjuvant treatment, completed the one year therapy: to endure flu-like symptoms and malaise for one year appeared to be the most important reason (in 60%). As this might be caused by levamisole as well as by 5FU both drugs were discontinued. Gradually patients withdrew from treatment. Toxic deaths were not encountered. In the Intergroup study compliance was similar (Moertel et al, 1990; Wolmark et al, 1993). Also in the schedules with the shorter 5FU plus leucovorin schedule (6 months) severe side effects were infrequent and consisted of life-threatening grade 4 mucositis (3%) or diarrhoea (4%), but they were not fatal (IMPACT, 1995, O'Connell et al, 1997). From the third cycle onwards the median dose of chemotherapy was 76% of the target dose, and only 35–45% of the patients received more than 80% of the target dose. Nevertheless, since the beneficial effect on survival seems to be equal, the shorter scheme of 5-FU plus leucovorin is generally preferred (O'Connell, 1998).

In summary, a positive effect of 5FU-based adjuvant treatment regimen in stage III colonic cancer has been demonstrated in various studies (IMPACT, 1995; Moertel et al, 1995; O'Connell et al, 1997) as in ours and is presently standard therapy. In stage II patients the available data are more limited and conflicting (Moertel et al, 1995; IMPACT, 1999; Mamounas et al, 1999). This study supports the hypothesis that adjuvant therapy might be equally effective in stage II colonic cancer. The effect of adjuvant systemic treatment in rectal cancer remains unclear.

### REFERENCES

Buyse M, Zeleniuch-Jacqoutte A and Chalmers TC (1988) Adjuvant therapy of colorectal cancer. Why we still don't know. *JAMA* **259**: 3571–3578

Fisher B, Wolmark N, Rockette H, Redmond C, Deutsch M, Wickerman DL, Fisher ER, Caplan R, Jones J, Lerner H, Gordon P, Feldman M, Cruz A, Legault-Poisson S, Wexler M, Lawrence W and Robidoux A (1988) Postoperative adjuvant therapy for rectal cancer: results from NSABP protocol R-01. *J Natl Cancer Inst* **80**: 21–29

 *© 2001 Cancer Research Campaign*

Frykholm GJ, Glimelius B and Pahlman L (1993) Preoperative or postoperative irradiation in adenocarcinoma of the rectum: final treatment results of a randomised trial and an evaluation of late secondary effects. *Dis Colon Rectum* **36**: 564–572

International Multicentre Pooled analysis of colon cancer trials (IMPACT) investigators (1995) Efficacy of adjuvant fluorouracil and folinic acid in colon cancer. *Lancet* **345**: 939–944

International Multicentre Pooled analysis of B2 colon cancer trials (IMPACT B2) investigators (1999) Efficacy of adjuvant fluorouracil and folinic acid in B2 colon cancer. *J Clin Oncol* **17**: 1356–1363

Krook JE, Moertel CG, Gunderson LL, Wieand HS, Collins RT, Beart RW, Kubista TP, Poon MA, Meyers WC, Mailliard JA, Twito DI, Morton RF, Veeder MH, Witzig TE, Cha S and Vidyarthi SC, (1991) Effective surgical adjuvant therapy for high-risk rectal carcinoma. *N Engl J Med* **324**: 709–715

Laurie JA, Moertel CG, Fleming TR Wieand S, Leigh JE, Rubin J, McCormack W, Gerstner JB, Krook JE, Mailiard J, Twito DI, Morton RF, Tschetter K and Barlow JF (1989) Surgical adjuvant therapy of large bowel carcinoma: an evaluation of levamisole and the combination of levamisole and fluorouracil: the North Central Cancer Treatment Group and the Mayo Clinic. *J Clin Oncol* **7**: 1447–1456

Mamounas EP, Rochette H, Jones J, Wieand S, Wickerman DL, Fisher B and Wolmark N (1997) Comparitive efficacy of adjuvant chemotherapy in patients with Dukes B versus Dukes C colon cancer: results from four NSABP adjuvant studies (CO1, CO2, CO3, CO4). *J Clin Oncol* **16**: 205

Marijnen CAM, Nagtegaal ID, Klein Kranenborg E, Hermans J, Van de velde CJH, Leer JWH and Van Krieken JHJM (2001) No downstaging after short-term preoperative radiotherapy in rectal cancer patients. *J Clin Oncol* **19**: 1976–1984

Moertel CG, Fleming TR, MacDonald JS, Haller DG, Laurie JA, Goodman PJ, Ungerleider JS, Emerson WA, Tormey DC, Glick JH, Veeder MH and Maillard JA (1990) Levamisole and fluorouracil for adjuvant therapy of resected colon carcinoma. *N Engl J Med* **322**: 352–358

Moertel CG, Fleming TR and MacDonald JS (1995) Fluorouracil plus levamisole as effective adjuvant therapy after resection of stage III colon carcinoma: a final report. *Ann Intern Med* **122**: 321–326

NIH consensus conference (1990) Adjuvant therapy for patients with colon and rectal cancer. *JAMA* **264**: 1444 –1450

O’Connell M, Maillard JA, Kahn MJ, Mac Donald JS, Haller DG, Mayer RJ and Wieand HS (1997) Controlled trial of fluorouracil and low-dose leucovorin given for 6 months as postoperative adjuvant therapy for colon cancer. *J Clin Oncol* **15**: 246–250

O’Connell M, Laurie JA, Kahn MJ, Fitzgibbons RJ, Erlichman C, Shepherd L, Moertel CG, Kocha R, Pazdur R, Wieand HS, Rubin J, Vukow AM, Donohue J, Krook JE and Figueredo A (1998) Prospectively randomized trial of postoperative adjuvant chemotherapy in patients with high-risk colon cancer. *J Clin Oncol* **16**: 295–300

Swedish Rectal Cancer Trial (1997) Improved survival with preoperative radiotherapy in resectable rectal cancer. *N Engl J Med* **336**: 980–987

Treurniet-Donker AD, van Putten WJL, Wereldsma JCJ, Bruggink EDM, Hoogenraad WJ, Ronkema JA, Snijders-Keilholz A, Meijer WS, Meerwaldt JH, Wijnmalen AJ and Wigger TH (1991) Postoperative radiation therapy for rectal cancer. An interim analysis of a prospective multicenter trial in the Netherlands. *Cancer* **67**: 2042–2048

Wolmark N, Rockette H, Fisher B, Wickerham DL, Redmond C, Fisher ER, Jone J, Mamounas EP, Ore L, Peterli N, Spurr CL, Dimitrov N, Romond EH, Surthrtland CM, Kardinal CG, Defusco P and Jochimsen P (1993) Benefit of leucovorin-modulated fluorouracil as postoperative adjuvant therapy for primary colon cancer: results from National Surgical Adjuvant Breast and Bowel Protocol C-03. *J Clin Oncol* **11**: 1879–1887

Wolmark N, Rockette H, Mamounas EP et al (1999) Clinical trial to assess the relative efficacy of Fluorouracil and leucovorin, Fluorouracil and levamisole, and Fluorouracil, leucovorin, and levamisole in patients with Dukes’B and C carcinoma of the colon: results from National Surgical Adjuvant Breast and Bowel Project C-04. *J Clin Oncol* **17**: 3553–3559

### ADDENDUM

### Participating hospitals with 10 or more patients per centre

Amersfoort, Eemland Hospital, de Lichtenberg
Amstelveen, Amstelveen Hospital
Amsterdam, Academic Medical Center
Amsterdam, Onze Lieve Vrouwen Gasthuis
Amsterdam, Netherlands Cancer Institute/Antoni van Leeuwenhoek Hospital
Amsterdam, Slotervaart Hospital
Arnhem, Diaconessen Hospital
Blaricum, Majella Hospital
Breda, Interconfessional Hospital De Baronie
Breda, St. Ignatius Hospital
Capelle a/d IJssel, IJsselland Hospital
Delft, Reinier de Graaf Hospital
Den Haag, Leijenburg Hospital
Den Haag, Rode Kruis Hospital
Dirksland, Van Weel Bethesda Hospital
Eindhoven, Catharina Hospital
Heemstede, Spaarne Hospital
Heerlen, De Wever Hospital
Hilversum, Reginal Hospital Hilversum
Hoorn, Westfries Gasthuis
Leiden, Academic Hospital
Leiden, Diaconessen Hospital
Leidschendam, St Antoiniushoeve
Purmerend, Waterland Hospital
Rotterdam, Daniel den Hoed Cancer Center
Rotterdam, Ikazia Hospital
Rotterdam, University Hospital Rotterdam Dijkigt
Spijkenisse, Ruwaard van Putten Hospital
Utrecht, Diaconessen Hospital
Utrecht, Overvecht Hospital
Utrecht, University Hospital
Tilburg, St Elisabeth Hospital
Tilburg, Maria Hospital
Venlo, St. Maartens Gasthuis
Vlaardingen, Regional Hospital Walcheren
Winterswijk, Regional Hospital Queen Beatrix
IJmuiden, Zeeweg Hospital

*© 2001 Cancer Research Campaign*
